# Universal newborn eye screening: a systematic review of the literature and review of international guidelines

**DOI:** 10.7189/jogh.12.12003

**Published:** 2022-10-21

**Authors:** Aeesha NJ Malik, Jennifer R Evans, Shuchita Gupta, Silvio Mariotti, Iris Gordon, Richard Bowman, Clare Gilbert

**Affiliations:** 1 Department of Clinical Research, London School of Hygiene and Tropical Medicine, London, UK; 2 Cochrane Eyes and Vision, Centre for Public Health, Queen’s University Belfast, Belfast, UK; 3 Department of Maternal, Newborn, Child and Adolescent Health (‎MCA), World Health Organization, Geneva, Switzerland; 4 Department of Noncommunicable Diseases, Eye and Vision Care, World Health Organization, Geneva, Switzerland

## Abstract

**Background:**

This systematic review assessed the effectiveness of universal screening for newborn eye abnormalities compared with no screening in improving infant vision and health outcomes.

**Methods:**

We searched CENTRAL (Cochrane Library), MEDLINE, Embase, Global Health, Global Index Medicus, clinical trials databases, and bibliographies of relevant articles. We included randomized and observational studies of all newborns, regardless of illness or risk factors, that compared universal screening for any eye abnormality by eight weeks of age with no universal screening. Two authors independently selected studies, extracted data, and evaluated the risk of bias. We used GRADE to assess the certainty of evidence. We also reviewed available recommendations on newborn eye screening.

**Results:**

Fourteen studies were identified but only three compared universal red reflex screening with no screening. Findings suggest that universal red reflex testing in maternity wards (MWs) may increase the number of newborns with congenital cataracts referred for eye care from MWs or well-baby clinics (WBCs) in the first year of life (risk ratio (RR) = 9.83, 95% confidence interval (CI) = 1.36-71.20; low certainty evidence). However, the effect of screening in WBC is uncertain (RR = 6.62, 95% CI = 0.87-50.09). The effect of MW or WBC screening on referral from any health care facility (MWs, WBCs, paediatrician clinic, other) in the first year is uncertain (MW screening: RR = 1.22, 95% CI = 0.63-2.39; WBC screening: RR = 0.97, 95% CI = 0.46-2.05). However, referral or surgery by 6 weeks of age may be higher with universal MW screening (early referral: RR = 4.61, 95% CI = 1.12-19.01; early surgery: RR = 8.23, 95% CI = 1.13-59.80; low certainty evidence). The effect of WBC screening on early referral and surgery is uncertain (early referral: RR = 1.98, 95% CI = 0.43-9.19; early surgery: RR = 3.97, 95% CI = 0.50-31.33; very low certainty evidence). Universal red reflex testing may increase clinical conjunctivitis (OR = 1.22, 95% CI = 1.01-1.47; low certainty evidence) but the effect on confirmed bacterial conjunctivitis is uncertain (OR = 1.20, 95% CI = 0.76-1.90; very low-certainty evidence). Nine guidelines recommended universal newborn eye screening using red reflex testing.

**Conclusions:**

Evidence supports the role of red reflex testing shortly after birth to increase early identification, referral, and surgery for congenital cataracts.

**Registration** PROSPERO (reference CRD42020180524).

In 2020, 1.44 million children aged 0-14 years were estimated to be blind or severely visually impaired [1]. Most blind children are either born blind from congenital conditions or become blind before the age of 5 years from acquired conditions [2,3].

The birth prevalence of eye anomalies in 21 European countries was 36.2 (95% CI = 34.6-37.9) per 100 000 births between 2011 and 2017. A third had congenital cataract [4]. The birth prevalence in low- and middle-income countries is likely to be higher due to a higher incidence of intrauterine infections, and consanguinity which increases the incidence of autosomal recessive conditions, such as cataract and glaucoma [5-7].

Treatable conditions which are potentially visually impairing, or which threaten life, need to be detected and managed early, as this gives better functional and survival outcomes. In high-income countries, surgery for dense bilateral congenital cataracts is usually performed 6-7 weeks after birth, as earlier surgery is associated with serious complications [8], and later surgery increases the risk of amblyopia [8,9]. In retinoblastoma, small lesions, if detected early, can be managed effectively, which preserves sight and improves survival [10-12]. In low and middle-income countries, mortality rates from retinoblastoma are far higher than in high-income countries, largely due to late presentation [13].

Newborn eye screening (NES) relies on examining the eyes and eliciting clinical signs. Methods include a torchlight examination of the external structures, and red reflex testing or wide-field digital imaging for internal structures. Red reflex testing, also called fundal reflex testing, refers to the reddish glow elicited within the pupil by light reflected from the retina when a light is shone directly into the eyes. Red reflex testing requires a handheld device, commonly a direct ophthalmoscope, and can be performed by anyone trained to deliver the test [14]. Wide-field imaging provides a view of all the intraocular structures but currently requires very expensive equipment which has limited availability. Assessment of visual acuity (ie, the ability to recognise small objects) is not possible in newborns.

In high-income countries, universal newborn eye screening (UNES) is the standard of care. However, in resource-constrained settings, UNES is often not included in newborn and child health policies or practiced routinely for all term healthy newborns. We conducted this systematic review to assess the evidence for the effectiveness of UNES to inform evidence-based guidelines for high, middle, and low-income countries. The primary objective was to assess the effect of UNES compared with no universal screening on newborn and infant vision and health outcomes. We also conducted a review of global, regional, and national guidelines to identify recommendations on UNES.

## METHODS

### Eligibility criteria

We included intervention studies and comparative observational studies (cohort/case-control/cross-sectional) if there was not adequate evidence from intervention studies. We included studies comparing universal screening for all newborns, irrespective of risk factors and complications compared to no universal screening. Studies enrolling only preterm infants were excluded. The intervention was defined as UNES within eight weeks of birth and the comparator was no UNES. The screening could be performed using any suitable ophthalmic device, ie, a torchlight to examine the eyelids and external eye, direct ophthalmoscope or other devices to elicit the red reflex, digital retinal imaging, and any other tests or devices which may be used for UNES in real-life settings. Studies in which screening was performed after eight weeks of age were excluded. We included studies from all country-income settings, conducted in health facilities or at home.

### Outcomes

The primary outcomes were the proportion of newborns identified with clinically significant eye conditions (see below) and referred or treated by eight weeks of age, age in months at referral, and outcomes of the clinical management of the eye condition in terms of health (eg, mortality) and ocular outcomes (eg, visual acuity) The secondary outcomes were adverse effects of eye screening, diagnostic test accuracy, and cost-benefit, cost-effectiveness, or cost estimates.

Clinically significant eye conditions were classified as, 1. conditions likely to be visually impairing which can be treated clinically or with optical correction, such as abnormalities of the eyelid(s) which obscure the pupil (eg, drooping eyelids (ptosis); congenital cataract; congenital glaucoma; retinoblastoma; inflammatory or vascular conditions on the retina; congenital anomalies, abnormally small eyes (microphthalmos) [15] and 2. conditions which cannot be treated but where early vision rehabilitation is required, such as absent iris (aniridia), coloboma (uveal defects), corneal opacities and most optic nerve and retina conditions (Table S1 in the **Online Supplementary Document**).

### Search strategy

An information scientist (IG) with experience in systematic reviews of eye conditions compiled the search terms with input from three of the study team who are paediatric ophthalmologists (AM/CG/RB). The electronic resources searched and the MEDLINE search strategy are provided in Appendix S1 and Appendix S2 in the **Online Supplementary Document**. Searches were conducted on April 13, 2020 and were updated on March 30 and September 8, 2021. No restrictions by language or publication year were applied. Conference abstracts were not searched. The MEDLINE search strategy was adapted to search the other resources listed (the Cochrane Library, Embase Ovid, Global Health Ovid, and Global Index Medicus).

Separate searches were not undertaken for the secondary outcomes of diagnostic test accuracy, cost-effectiveness, cost-benefit, or costing. These outcomes were documented in this review if they were reported in included studies or were identified in the search.

### Selection of studies

Two experts independently reviewed the titles and abstracts for eligibility and full texts of all potentially relevant articles were reviewed independently for inclusion (AM, CG). Discrepancies at any stage were resolved through discussion. Articles in languages other than English were either sent to a bilingual ophthalmologist, with a list of questions to ascertain whether they fulfilled the inclusion criteria, and one was translated using Google translate. Articles excluded at this stage were listed giving a reason for exclusion (Appendix S3 in the **Online Supplementary Document**).

### Data extraction

Data were extracted independently by two review authors (AM, CG) using a pre-piloted form. Findings were compared and discrepancies were resolved by discussion. The data extracted are detailed in Appendix S4 in the **Online Supplementary Document**. Authors of included studies were contacted, when necessary, for clarification or to provide additional data.

### Risk of bias assessment

Risk of bias assessment was undertaken independently by three authors (JE, CG, AM) using the ROBINS-I tool for non-randomized trials of interventions for studies included in the quantitative analysis [16] Any discrepancies were resolved by discussion.

### Analysis

Data from comparative studies were presented using appropriate measures of effect, where possible (eg, risk ratios, odds ratios). Adjusted estimates were used for observational studies when reported by the included studies. Relevant effect measures (risk ratio with 95% CI) and sensitivity, specificity, and positive predictive values (with 95% CI) were calculated using Revman 5.4 [17] and Medcalc [18,19]. We planned to undertake a meta-analysis if the heterogeneity in study designs, interventions, and outcomes allowed. The certainty of the evidence was assessed using the GRADE approach [20].

### Review of guidelines

Databases of guidelines and websites were searched for NES guidelines (Appendix S1 in the **Online Supplementary Document**). The criteria for selection were any newborn, child health, or eye health guidelines that referred to NES. The target population, setting, and interventions were the same as for the systematic literature review.

## RESULTS

**Figure 1:**
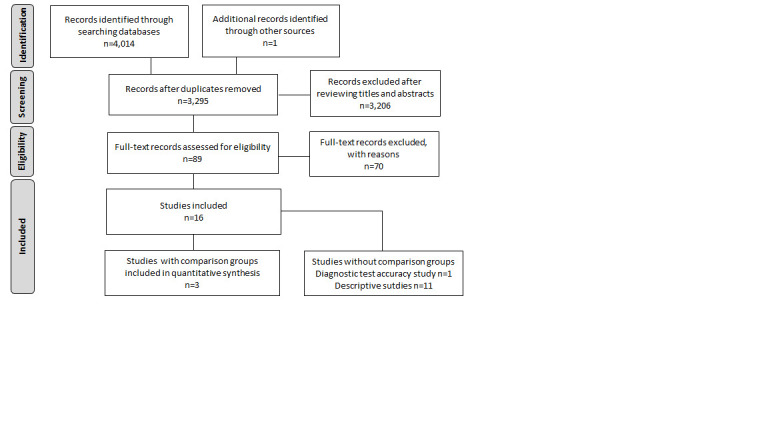
PRISMA flow diagram.

We identified 14 studies on UNES involving 1 018 467 infants (Table S2a, Table S2b and Table S3 in the **Online Supplementary Document**). Only three of these studies compared UNES to no screening. Two of these three studies addressed the primary outcomes [21,22], and one addressed secondary outcomes [23] and were included in the quantitative analysis. (**Figure 1**, Table S3 in the **Online Supplementary Document**).

Of the remaining 11 studies, one study was a diagnostic test accuracy study [24], while the rest (10 studies) were descriptive, and did not have a comparator group. These studies described the proportion and type of eye conditions identified by NES. We have summarized the results from these studies in Table S4a and Table S4b in the **Online Supplementary Document**. All the studies were undertaken in high-income or upper-middle-income settings.

All the three studies included in the quantitative analysis were undertaken in high-income countries (two in Sweden [21,22] and one in Israel [23]. The risk of bias assessments is summarized in Appendix S5 in the **Online Supplementary Document**.

### Primary outcomes

None of the included studies reported visual acuity outcomes or neonatal/infant mortality or neurodevelopment outcomes.

Two included studies from Sweden, that compared UNES with no screening, reported the proportion of infants referred or treated by eight weeks of age [21,22]. The first study compared two regions (Region 1 and Region 2) where UNES using red reflex testing was undertaken in different locations (maternity wards or well-baby clinics) with another region (Region 3) where there was no UNES. The study period was from 1992 to 1998. The population covered by the three regions included an estimated 396 000 newborns. Region 1 established universal red reflex testing with an external eye examination in the maternity ward during the first few days after birth. Region 2 used the same screening test performed in well-baby clinics at around 6 weeks of age while Region 3 did not have UNES. The second study added national data from 2007 to 2009 to the first study, when UNES was a routine procedure in 90% of maternity wards (estimated total population 328 523 newborns). No details are provided on who performed the screening.

Both studies compared UNES using red reflex testing with no screening. Both were facility-based, retrospective cohort studies. Data on the outcomes were extracted by the study authors from a Scandinavian register (PECARE) of children operated for unilateral or bilateral cataract by 8 years of age in Scandinavian countries. The data were limited to children who had cataract surgery during the first year of life in Sweden, after excluding children with traumatic cataract.

The studies reported the proportion of newborns referred or operated on by six weeks of age. The babies could be referred from maternity wards, well-baby clinics, or any other facility (eg, paediatric clinic). The studies reported data separately for babies screened in maternity wards, well-baby clinics, or who were referred from any location (maternity wards, well-baby clinics, or any other facility) in the first year of life and during the first six weeks after birth. A more detailed breakdown of the data was provided by the author (Gunilla Magnusson; personal communication). Both studies were considered to be at serious risk of bias (Appendix S5 in the **Online Supplementary Document**).

The data suggest that UNES using red reflex testing in maternity wards may increase the proportion of newborns referred with congenital cataract from maternity wards or well-baby clinics in their first year compared with no screening (one study, 394 438 infants; RR = 9.83, 95% CI = 1.36-71.20; low certainty evidence), but the effect of well-baby clinic screening on this outcome is uncertain (one study, 215 347 infants, RR = 6.62, 95% CI = 0.87-50.09; very low certainty evidence) (**Table 1**).

**Table 1:** Summary of findings table for universal newborn screening vs no screening: proportion of newborns with congenital cataract referred within the first year of life (outcome a and b), and referred and operated by six weeks of age (outcome c and d) [21,22]

	Studies (participants)	Event n/N			Effect (95% CI)			Certainty of evidence (GRADE)
**Universal screening**		**No universal screening (Region 3, 1992-98)**	**Relative risk**		**Absolute risk, per 1000 neonates**
**Outcome A) newborns with congenital cataract referred from maternity wards or well-baby clinics by the age of one year**								
Maternity ward screening (national data; 2007-09)*	1 (n = 394 438)	49/328 523		1/65 915	9.83 (1.36-71.20)		0 fewer per 1000 (from 0 fewer to 1 more)	Low^†^
Well-baby clinic screening (Region 2; 1992-98)	1 (n = 215 347)	15/149 432		1/65 915	6.62 (0.87-50.09)		0 fewer per 1000 (from 0 fewer to 1 more)	Very low^‡^
Outcome B) newborn with congenital cataract referred from any health care facility* by the age of one year								
Maternity ward screening* (national data; 2007-09)	1 (n = 394 438)	61/328 523		10/65 915	1.22 (0.63-2.39)		0 fewer per 1000 (from 0 fewer to 0 fewer)	Very low^§^
Well-baby clinic screening (Region 2; 1992-98)	1 (n = 215 347)	22/149 432		10/65 915	0.97 (0.46-2.05)		0 fewer per 1000 (from 0 fewer to 0 fewer	Very low^§^
Outcome C) newborns with congenital cataract referred by 6 weeks of age from any health care facility*								
Maternity ward screening* (national data; 2007-09)	1 (n = 394 438)		46/328 523	2/65 915	4.61 (1.12-19.01)	0 fewer per 1000 (from 0 fewer to 1 more)		Low^†^
Well-baby clinic screening (Region 2; 1992-98)	1 (n = 215 347)		9/149 432	2/65 915	1.98 (0.43-9.19)	0 fewer per 1000 (from 0 fewer to 0 fewer)		Very low^‡^
Outcome D) newborn with congenital cataract operated by 6 weeks of age referred from any health care facility*								
Maternity ward screening* (National data; 2007-09)	1 (n = 394 438)		41/328 523	1/65 915	8.23 (1.13-59.80)	0 fewer per 1000 (from 0 fewer to 1 more)		Low^†^
Well-baby clinic screening (Region 2; 1992-98)	1 (n = 215 347)		9/149 432	1/65 915	3.97 (0.50-31.33)	0 fewer per 1000 (from 0 fewer to 0 fewer)		Very low^‡^

CI – confidence interval

*Maternity ward, well-baby clinics, paediatrician clinic, or other.

†Downgraded by two levels due to very serious risk of bias.

‡Downgraded by more than three levels due to very serious risk of bias and very serious imprecision.

§Downgraded by three levels due to very serious risk of bias and serious imprecision.

It is uncertain whether UNES using red reflex in maternity wards or the well-baby clinics has an effect on the proportion of newborns with congenital cataract referred from any health facility (maternity ward, well-baby clinic, by a paediatrician, or other) in the first year compared with no screening (one study, 394 438 infants; RR = 1.22, 95% CI = 0.63-2.39 for maternity ward screening and RR = 0.97, 95% CI = 0.46-2.05 for well-baby clinic screening; very low certainty evidence).

UNES using red reflex in maternity wards may increase the proportion of newborns with congenital cataract referred early, ie, by 6 weeks of age from any health care facility compared with no screening (one study, 394 438 infants; RR = 4.61, 95% CI = 1.12-19.01), but the effect of universal screening in well-baby clinics on this outcome is uncertain (one study, 215 347 infants, RR = 1.98, 95% CI = 0.43-9.19; very low certainty evidence) (**Table 1**).

UNES using red reflex testing in maternity wards may increase the proportion of newborns with congenital cataract referred from any health care facility who are operated early, ie, by 6 weeks of age, compared with no screening (one study, 394 438 infants; RR = 8.23, 95% CI = 1.13-59.80; low certainty evidence). It is uncertain whether universal screening in well-baby clinics has any effect on the proportion of newborns with congenital cataract operated by 6 weeks of age compared with no screening (one study, 215 347 infants, RR = 3.97, 95% CI = 0.50-31.33; very low-certainty evidence).

### Secondary outcomes

#### Adverse effects

One hospital-based, before-and-after study in a maternity unit in Israel, reported adverse events associated with UNES using red reflex testing [23] The study evaluated whether introducing red reflex testing of newborns by a physician increased the proportion of newborns with clinical conjunctivitis and with microbiologically confirmed bacterial conjunctivitis. Pre-intervention data (2008/2009) were compared with data post-intervention data (2010/2011) among a total of 18 872 newborns. This study was judged to be at serious risk of bias due to confounding (Appendix S5 in the **Online Supplementary Document**).

Universal screening using red reflex testing may increase the occurrence of clinical conjunctivitis compared with no screening (OR = 1.22, 95% CI = 1.01-1.47; low certainty evidence), but the effect on confirmed bacterial conjunctivitis is uncertain (OR = 1.20, 95% CI = 0.76-1.90; very low-certainty evidence) (**Table 2**).

**Table 2:** Universal red reflex screening vs no screening: adverse effects [23]

Neonatal outcomes	Studies (neonates)	Effect: odds ratio (95% CI)	Effect: Absolute risk, per 1000 neonates (95% CI)	Certainty of evidence (GRADE)
Clinical conjunctivitis	1 (n = 18 870)	1.220 (1.01-1.47)	5 more per 1000 (from 0 fewer to 10 more)	Low*
Confirmed bacterial conjunctivitis	1 (n = 18 870)	1.20 (0.76-1.90)	1 more per 1000 (from 1 fewer to 3 more)	Very low†

CI – confidence interval

*Downgraded by two levels due to very serious risk of bias.

†Downgraded by three levels due to very serious risk of bias and serious imprecision.

#### Diagnostic test accuracy

One study in China of 7641 consecutive, healthy, full-term newborns in a large maternity unit assessed diagnostic test accuracy (DTA) of red reflex testing with an external examination of the eye using torchlight 2-4 days after birth [24] The gold standard was dilated anterior and posterior segment wide-field imaging, which was performed immediately after red reflex testing. Those with undiagnosed ocular abnormalities subsequently underwent indirect ophthalmoscopy and ultrasound. All procedures were conducted by a paediatric ophthalmologist. Data were extracted on clinically significant eye conditions only by the review authors for analysis. This study was considered to be at high risk of bias as the same examiner performed both tests which were undertaken sequentially.

For all clinically significant eye conditions the sensitivity of red reflex testing was 3.5% (0.4%-12.1%) with a specificity of 96.0% (95.6%-96.5%) and a positive predictive value of 0.7% (0.2%-2.6%). The sensitivity was 66.7% (9.4%-99.2%) for anterior segment conditions only, and 0% for posterior segment conditions only.

### Review of recommendations

Eight national publications were identified which recommend UNES (Appendix S6 in the **Online Supplementary Document**, **Table 3**). Four were published by professional associations (in Canada, the United States of America (3 documents)), and four were produced by Ministries of Health or government bodies (in Canada, United Kingdom, India, and New Zealand). All the recommendations were based on expert consensus and used limited direct evidence. We also identified a training manual from the World Health Organization’s European Office, which included NES as part of the newborn assessment in the Integrated Management of Childhood Illness. A relevant policy document could not be identified.

**Table 3:** Recommendations for newborn eye screening in guidelines / preferred practice recommendations

Organization, country, title	Year/income	Age at screening	Screening test	Person conducting screening/location of screening
American Academy of Ophthalmology. Vision Screening for Infants and Children. A joint statement of the American Association for Pediatric Ophthalmology and Strabismus, and the American Academy of Ophthalmology.	2013/High	Newborn	• RRT • “General eye health”	• Ophthalmologist, pediatrician, family doctor, trained health professional • “Newborn nursery”
American Academy of Ophthalmology. Paediatric Eye Evaluations Preferred Practice Pattern	2017/High	Newborn-6 mo	• RRT with ophthalmoscope • External eye examination • Pupil examination	• Trained physicians, nurses, others • Primary care/community health professional
American Academy of Pediatricians. Policy document with American Academy of Ophthalmology/American Association for Pediatric Ophthalmology and American Association of Orthoptists Red reflex examination in neonates, infants, and children.	2016/High	Newborn-6 mo	• RRT • External eye examination • History	• Paediatricians
Public Health England. Newborn and infant physical examination (NIPE) screening programme handbook. Updated 27 August 2019.	2016/2019/High	Newborn (within 72 h) and 6-8 weeks	• RRT with ophthalmoscope • External eye examination • History	• Paediatric doctor, family doctor • Maternity unit, well-child visit
Joint Clinical Practice Guideline Expert Committee of the Canadian Association of Optometrists and the Canadian Ophthalmological Society. Evidence-based clinical practice guidelines for the periodic eye examination in children aged 0-5 y in Canada.	2019/High	Newborn - 3 mo	• RRT with ophthalmoscope • External eye examination	• Primary care provider/non ophthalmological personnel • Well baby visit
Government of Canada, First Nations and Inuit Health Branch. Clinical Practice Guidelines for Nurses in Primary Care, Chapter 8: Pediatric and Adolescent Care – Eyes.	2010/High	Newborn - 3 mo	• RRT with ophthalmoscope • External eye examination • Corneal reflex for strabismus	• Nurses • Primary care
Ministry of Health, New Zealand. Well Child / Tamariki Ora Programme Practitioner Handbook: Supporting families and whānau to promote their child’s health and development. Revised 2014.	2014/High	Newborn - 7 d	• RRT with ophthalmoscope • External eye examination	• “Practitioner trained to use a direct ophthalmoscope”
Ministry of Health and Family Welfare, Government of India, Rashtriya Bal Swasthya Karyakram. Guidelines for universal eye screening in newborns including retinopathy of prematurity.	2017/Lower middle	Newborn - 48-72 h	• RRT with ophthalmoscope • External eye examination • White reflex with torch • History	• Medical officers, pediatricians, nurses • Place of delivery/neonatal units
World Health Organization, Office for Europe. Effective Perinatal Care (EPC) Training Package. 2nd edition [25]	2015/Most high	Newborn, day 3, 7-14, week 6	• RRT with ophthalmoscope • External eye examination	• Doctors, nurses, midwives

RRT – red reflex test

All documents recommended red reflex testing and external eye examination, with four also recommending taking a history. One also recommended a torch examination to elicit a white reflex (eg, for dense cataract). All the guidelines recommended testing shortly after birth, and the age range varied from within days of birth to 6 months, but most (78%) recommended before three months of age. A wide range of personnel was recommended to carry out the examination, including ophthalmologists, paediatricians, family doctors, primary care providers, nurses, and midwives. The location of screening, where specified, was at the place of birth or in a primary care setting. All guidelines, apart from the training curriculum, recommended referral for infants with an abnormal red reflex, with five specifying referral to a tertiary eye care facility or an ophthalmologist.

## DISCUSSION

Our review of three comparative studies involving over 780 000 newborns suggests that UNES using red reflex testing in maternity wards, ie, shortly after birth, may increase the number of newborns with congenital cataract who are referred and operated on early, ie, by six weeks of age. The direction of effect was similar for universal screening conducted in well-baby clinics but the certainty of the evidence was very low. There is no evidence that red reflex testing increases adverse events though the data were very limited.

One study assessed the diagnostic test accuracy of red reflex testing suggests that it has a sensitivity of 67% for clinically significant conditions in the anterior segment and a specificity of 96% for anterior plus posterior segment conditions. We identified one study which estimated the cost-effectiveness of adding a second screening site, which was not included in the review as it did not assess screening vs no screening [26]. We may have missed other studies of cost-effectiveness and DTA as specific searches were not conducted for these outcomes.

All three studies included in the quantitative analysis [21-23] were undertaken in high-income countries and the DTA study was undertaken in China, an upper-middle-income country [24]. The studies from Sweden suggest that UNES using red reflex testing shortly after birth may lead to higher detection of congenital cataract, and earlier referral and uptake of cataract surgery. The value of UNES is also supported by a surveillance study of infantile cataract (ie, present at birth or developed by one year of age) in the United Kingdom in which almost half the children had been detected by red reflex testing shortly after birth or at 6-8 weeks [27]. These studies highlight the importance of UNES even in high-income countries where parents are well educated and health services are generally accessible and of high quality. Early referral and treatment are especially relevant in low-income settings as children with early-onset cataract often present very late for surgery [28], which compromises visual outcomes [29-31]. Children with retinoblastoma also often present very late, with local extraocular spread or widespread dissemination, which limits their life expectancy [32]. In these settings young children often have limited access to services after birth, and parents can be poorly educated and not aware of the signs of clinically significant eye conditions. UNES will enable early detection and timely referral of newborns with eye abnormalities in these settings.

None of the studies had the longer-term objective of evaluating whether UNES leads to better clinical outcomes. However, evidence from the case series shows that early referral and management leads to better outcomes than late treatment [8,9]. This is an important consideration in low-resource settings because screening should be followed by appropriate diagnosis and management to ensure optimal outcomes. This requires well-equipped tertiary-level eye care facilities for young children, with a trained team led by a pediatric ophthalmologist. Diagnostic and surgical equipment and consumables for cataract surgery in young children are needed, including vitrectomy machines, high-power intraocular lenses, and small spectacle frames [33].

Our review suggests that red reflex testing is reasonably sensitive (67%, 95% CI = 9%-99%) for clinically significant anterior segment conditions, which is acceptable for screening purposes in most settings. However, we identified only one high-quality study [24] We identified two other DTA reviews of red reflex testing in newborns, which report lower sensitivities (23% and 17.5%) [34,35]. The high specificities reported (98% and 97.5%) are comparable to the study we report. The variation in sensitivities may reflect differences in the primary studies in terms of methodology and sample size, and all eye conditions were included in these two reviews, regardless of their clinical significance.

In ten of the descriptive studies, the proportion of newborns identified with clinically significant eye conditions in different contexts varied considerably regardless of the screening method used (red reflex testing or widefield imaging) (Table S4a and Table S4b in the **Online Supplementary Document**). While this may reflect true differences in the prevalence of these conditions in different populations, it may also reflect variation in the skills of those performing the test. Selection bias in the included studies is also likely, as sicker newborns, such as those with cataract from intrauterine infection, may have had a longer inpatient stay than healthy newborns in some settings, and so would still be inpatients if NES was performed a few days after birth. Retinoblastoma was more likely to be detected by imaging than by red reflex testing, as widefield imaging can detect small and peripheral lesions in addition to central tumours, which can also be detected by red reflex testing.

The studies informing the primary outcomes in this review only provided data on congenital cataract. The evidence was also primarily on red reflex testing, with limited data on other modalities such as wide-field digital imaging, which is likely to be more effective than red reflex testing for conditions such as retinoblastoma. However, it might be premature to propose wide-field imaging for UNES owing to the limited evidence on effectiveness and high cost of the device. In addition, current devices need contact with the cornea, which has implications for the skills and training required.

Our review of clinical guidelines suggests a high level of consensus among professional associations and pediatric and ophthalmology experts on UNES using red reflex testing. There are several reasons why recommendations are in place despite limited published evidence of effectiveness. First, case series show the benefits of early management of clinically significant eye conditions which can be present at birth, such as cataract and retinoblastoma, in terms of visual acuity and complications and survival, respectively. Second, red reflex testing is safe with minimal harm as it is a non-contact test that only takes a few minutes to perform. Third, many different health care professionals can be trained to become highly competent at red reflex testing, making it feasible in most settings. Lastly, several different devices are available for red reflex testing at a range of prices, which makes the equipment relatively affordable.

Our search did not identify any guidelines on UNES for any low-income and only one European country, the United Kingdom. In Europe, red reflex testing is included in the training materials for newborn care in the World Health Organization’s Integrated Management of Childhood Illness. This probably explains why a study of 35 European countries (2013-4) showed that 28 (80%) had a national UNES programme and the remaining seven had regional screening programmes [36]. In Tanzania, following a study that evaluated an eye care training module that included red reflex testing by primary child health care workers, eye screening was included in the curriculum of the Integrated Management of Childhood Illness. More than 3000 staff have since been trained to perform eye screening in newborns and young children [37]. Another study in Tanzania assessed the feasibility of different devices for red reflex screening, including a novel, low-cost direct ophthalmoscope [38]. Primary health workers found the device easy to use and having a device increased their sense of professionalism. Subsequently, large-scale red reflex testing resulted in a large number of children aged 0-5 years being detected with cataract in the community, demonstrating the feasibility of red reflex testing in low-income settings [39]. Another advantage of this device is that it can be attached to a mobile phone, and images of the reflex can be captured to educate parents or they can be forwarded for a second opinion.

The limited published evidence highlighted by this review is a major limitation of the study. Two studies were an evaluation of a screening programme in Sweden and the third was a before and after study; both of these study designs are subject to bias and the influence of confounding and external factors which weaken the level of evidence. The lack of evidence may reflect the fact that UNES is standard practice in many high-income countries and further studies of effectiveness may not be considered ethical. It is, therefore, unlikely that further effectiveness studies will be undertaken. New screening devices are also being developed, including a device that uses infrared light [40], and these need to be compared with existing devices in terms of DTA, ease of use, image quality, and cost. Further studies are needed in resource-poor settings to assess the feasibility, acceptability, and cost of including red reflex testing as an integral component of the standard newborn assessment in all facilities delivering babies.

## Additional material


Online Supplementary Document
