# Supplementary material for: Universal newborn eye screening: a systematic review of the literature and review of international guidelines
Source: J Glob Health. 2022 Oct 21;12:12003. doi: 10.7189/jogh.12.12003 (PMC9586142; doi:10.7189/jogh.12.12003)
Supplement: Online Supplementary Document [file jogh-12-12003-s001.pdf]

Supplementary Document

Table S1. Example of clinically significant and insignificant eye conditions from Tang et al(1)

Significant eye conditions	N	N100,000
Retinoblastoma	5	2.5
Cataract	39	19.9
Choroidal coloboma	95	48.4
Primary hyperplastic vitreous	25	12.7
Familial exudative vitreo-retinopathy	215	109.6
**Insignificant eye conditions**		
Retinal haemorrhage grade 3	12657	6454
ROP/ROP-like	114	58.1
Sub-conjunctival haemorrhage	254	129.5
Abnormal fundus pigment	1480	747.7
Persistent pupil membranes	105	53.5
Retinal dysplasia	191	97.4
Albinotic fundus	31	15.8
Venous tortuosity	228	116.3
Exudative changes ? diagnosis	1238	631.3
Retinal vessel hypoplasia	114	58.1
Abnormal retinal vessels	456	232.5
Other	391	199.4

Appendix S1. Information Sources

Electronic databases searched for systematic review:

Academic databases

- The Cochrane Library https://www.cochranelibrary.com/
- MEDLINE Ovid https://www.ovid.com/product-details.901.html
- Embase Ovid https://www.ovid.com/product-details.903.html
- Global Health Ovid https://www.ovid.com/product-details.30.html
- Global Index Medicus https://www.who.int/library/about/The_Global_Index_Medicus/en/

Trials registers

- US National Institutes of Health Ongoing Trials Register ClinicalTrials.gov www.clinicaltrials.gov
- World Health Organization (WHO) International Clinical Trials Registry Platform (ICTRP) www.who.int/ictrp

Grey literature

- OpenGrey http://www.opengrey.eu/
- PQDT Open Access Dissertations &Theses https://pqdtopen.proquest.com/search.html

Searching other sources

The bibliographies of all included studies and guidelines were reviewed.

Electronic databases searched for guidelines review:

The following databases were searched to identify guidelines of relevance:

- International Council of Ophthalmology and regional societies of ophthalmology
- Australian National Health and Medical Research Council clinical practice guidelines
- Canadian Medical Association Infobase of Clinical Practice Guidelines
- ECRI Guidelines Trust Database
- eGuidelines
- Guideline Central
- Guidelines International Network (GIN)
- National Institute for Clinical Excellence (UK) – NICE
- Scottish Intercollegiate Guidelines Network (SIGN)
- Trip Database
- US Preventive Services Task Force Guidelines
- WHO Guidelines

The bibliographies of papers included in the systematic review were also searched.

Appendix S2. MEDLINE search strategy

1. Neonatal Screening/
2. ((neonat$ or newborn$ or new born or new borns or newly born or baby$ or babies or infant or infants) adj2 screen$).tw.
3. ((neonat$ or newborn$ or new born or new borns or newly born or baby$ or babies or infant or infants) adj2 test$).tw.
4. ((neonat$ or newborn$ or new born or new borns or newly born or baby$ or babies or infant or infants) adj2 exam$).tw.
5. ((neonat$ or newborn$ or new born or new borns or newly born or baby$ or babies or infant or infants) adj2 assess$).tw.
6. or/1-5
7. Cataract/
8. cataract$.tw.
9. Glaucoma/
10. glaucoma$.tw.
11. Retinoblastoma/
12. retinoblastoma$.tw.
13. (retina$ adj3 (cancer$ or neoplas$ or tumor$ or tumour$ or malignan$ or carcinoma$ or adenocarcinoma$)).tw.
14. Eye Diseases/cn [Congenital]
15. exp Eye Abnormalities/
16. ((eye or ocular or vision or visual) adj2 (abnormal$ or patholog$)).tw.
17. (buphthalmos$ or buphthalmia$ or hydrophthalmos$).tw.
18. Eyelid Diseases/
19. Blepharoptosis/
20. (ptosis or blepharoptos$).tw.
21. Blepharophimosis/
22. blepharophimos$.tw.
23. (microphthalmos or nanophthalmos).tw.
24. leukoma.tw.
25. (congenital adj3 squint$).tw.
26. or/7-25
27. 6 and 26
28. Infant, Newborn/
29. Infant/
30. (neonat$ or newborn$ or new born or new borns or newly born or baby$ or babies or infant or infants).tw.
31. or/28-30
32. Reflex, Pupillary/
33. red reflex$.tw.
34. white reflex$.tw.
35. leukocoria.tw.
36. pupillary exam$.tw.
37. (cornea$ adj2 light adj2 reflex$).tw.
38. (photo adj1 refractometer).tw.
39. photorefraction.tw.
40. (Hirschberg adj2 (test$ or ratio$)).tw.
41. flashlight.tw.
42. Ophthalmoscopy/
43. ophthalmoscop$.tw.
44. Retinoscopy/
45. Retina/dg [Diagnostic Imaging]
46. retinoscop$.tw.
47. (wide adj1 field adj1 digital adj1 imag$).tw.
48. Vision Screening/

49. Diagnostic Techniques, Ophthalmological/
50. or/42-49
51. (retinopathy of prematurity or ROP).ti.
52. 50 not 51
53. 32 or 33 or 34 or 35 or 36 or 37 or 38 or 39 or 40 or 41 or 52
54. 31 and 53
55. (newborn adj2 eye adj2 (screen$ or exam$ or disease$)).tw.
56. ((neonatal or perinatal) adj2 (eye$ or ophthalmic or ocular) adj2 (screen$ or exam$ or imag$)).tw.
57. (universal adj2 (eye$ or ophthalmic or ocular) adj2 (screen$ or exam$ or imag$)).tw.
58. or/55-57
59. 27 or 54 or 58
60. exp case reports/
61. case report$.tw.
62. 60 or 61
63. 59 not 62
64. (mouse or mice or rat or rats or dog or dogs or monkey or monkeys).tw.
65. 63 not 64
66. limit 65 to (address or autobiography or bibliography or biography or clinical trial, veterinary or clinical trials, veterinary as topic or comment or editorial or "expression of concern" or festschrift or interactive tutorial or interview or lecture or legal case or legislation or letter or news or observational study, veterinary or periodical index or personal narrative or portrait or video-audio media or webcast)
67. 65 not 66

Appendix S3. Reasons for exclusion after full text review

Wrong study design	17
Wrong population	13
General review	11
Wrong outcomes	10
Small sample size	9
Wrong intervention	5
Wrong indication	1
Very old publication	1
Foreign language	1
Study protocol	1
Inadequate data	1
**TOTAL**	**70**

Appendix S4. Data variables extracted

The following data were extracted for all studies: author, study dates, country and income group, and study population and subgroups if relevant. Findings were reported according to the study design as follows:

- Observational studies of screening: data were extracted on the number of newborns eligible for screening; method, location and personnel screening; number screened by age, sex, ethnic group and subgroup. Data were extracted on the number of infants with clinically significant eye conditions overall and for most three most common eye conditions (cataract, glaucoma and retinoblastoma) and other significant conditions, and the proportions per 100,000 were calculated. Age at clinical management, and/or referral as an indirect measure, were extracted if available. Adverse events associated with screening were documented.
- Diagnostic test accuracy studies: data were extracted which would allow the construction of two by two tables for clinically significant eye conditions only.
- Other study designs, such as cost effectiveness studies. Data were extracted, as relevant.

The following data were extracted from the review of guidelines: organization and country; date of guideline; conditions being screened for; tests recommended and the quality of the evidence for the recommendation; age group to be included; who should perform the screening/examination, when this should occur and where this could be conducted.

Table S2a. Details of included studies

Commented [JE1]: Seem to be 14 studies here not 15 as in the abstract or 16 in the next table.

Now correct: 14 publications reported 15 outcomes/findings

	Study / country/status*	Date conducted	Type of study	Study setting	Study population / ethnic group/% female	Exclusions	Number of newborns	Age at screening
	**Included in quantitative analysis**							
1	Magnusson 2003, Sweden, H(2)	1992 to 1998	Case only study to compare three regions	Maternity wards and well-baby clinics	All births/ no data/no data	Not specified	Total births in study populations 396,000	Two groups: within first or 6 weeks
2	Magnusson 2013, Sweden, H(3)	Jan 2007 to Dec 2009	Cases only study to compare national data and data from 3 regions in 2003 study	Maternity wards and well-baby clinics	All births/no data/no data	Not specified	Total births in study population 328,523	Not specified
3	Ulanovsky 2015, Israel, H(4)	2008 to 2009 and 2010 to 2011	Cross-sectional before-& after study	Well-baby nursery	All newborns/no data/no data	≤35 gestation; admitted to NICU; sepsis; congenital abnormalities of nasolacrimal duct/no data/52%	18,872	Within first week
	**Diagnostic test accuracy**							
4	Sun 2016,** China, UM(5)	Sept 2014 to March 2015	Cross-sectional	Maternity and child hospital	All full-term newborns	Preterm (<37 weeks); admitted to neonatal intensive care; ocular deformity/no data/48%	7,641	Within first week
	**Descriptive studies – red reflex testing**							
5	Baldino 2019, Brazil, UM(6)	Jan 2014 to Jan 2018	Cross-sectional with nested case-control	Tertiary hospital maternity unit	Full-term newborns/no data/ (51% control group)	In intensive or semi intensive care or incomplete medical records	11,833	Within first week
6	Cagini 2017, Italy, H(7)	Jan 2012 to Dec 2014	Cross-sectional;	Regional birth centres	All births/no data	Not specified	22,884	Within first week
7	Eventov-Friedman 2010, Israel, H(8)	2007 to 2008	Cross-sectional	General hospital	All newborns/no data/no data	Not specified	11,500	Within first week
8	Yazgan 2012, Turkey, M(9)	Jan 2007 to Jan 2010	Cross-sectional	Paediatric clinic in hospital	All newborns/no data/no data	Not specified/no data/50%	2,718	Within 8 weeks
	**Descriptive studies – wide-field imaging**							
9	Goyal 2018, India, LM(10)	March 2014 to Oct 2015	Cross-sectional / (Costing)	General hospital	"Apparently healthy" newborns/no data/52%	Babies >4 weeks/too sick for screening, at discretion of pediatrician	1,152	Within first 28 days
10	Li 2013, China, UM(11)	May 2010 to June 2011	Cross-sectional	Maternal and child hospital	Full-term newborns/no data/50%	<2500g; systemic disease; Apgar score <7; mother with infectious disease	3,573	Within first week

11	Li 2017, China, UM(12)	March 2010 to February 2014	Cross-sectional	Maternal and child hospital	Full-term newborns/no data/48%	As above	15,284	Within first 28 days
12	Ozkurt 2018, Turkey, UM(13)	June 2013 to August 2014	Cross-sectional	General hospital; maternity and child health hospital	Newborns in hospital in rooming-in unit and neonatal intensive care	Not specified/no data/no data	In neonatal unit: 800, in rooming-in unit, 558	Not specified
13	Tang 2018, China, UM(1)	May 2009 to June 2017	Cross-sectional with comparison of term vs preterm neonates	Eight maternal and child hospitals	All newborns whose parents could afford digital imaging	Term (>34 weeks and >2000g); preterm (≤34 weeks or ≤2000g)/no data/56%	199,851: 196,108 not preterm; 3,743 preterm	Within 6 weeks
14	Vinekar 2015, India, LM(14)	September 2012 to March 2013	Cross-sectional	General public hospital with maternity unit	Full-term newborns/no data/no data	Birthweight ≤2000g/no data/45%	1,021	Within first week

*H = high income; UM = upper middle income; LM= lower middle income; L=low income; DTA = diagnostic test accuracy;

**Reported diagnostic test accuracy and the clinical findings of screening

NIPE: Newborn and Infant Physical Examination; UK programme; ROP: retinopathy of prematurity; Full term: gestational age of 37 weeks or older; DTA: diagnostic test accuracy

Table S2b. Details of included studies

Study	Examination	Details	Who did the screening examination	Examination to confirm abnormality
**Included in quantitative analysis**				
Magnusson 2003	Red reflex test	Direct ophthalmoscope	"Doctors and nurses"	Referral to an ophthalmologist implied
Magnusson 2013	Red reflex test	Direct ophthalmoscope	Paediatrician	Referral to ophthalmologist implied
Ulanovsky 2015	Red reflex test	Direct ophthalmoscope	Physician	NA
**Diagnostic test accuracy**				
Sun 2016**	Comprehensive eye exam	External eye exam, dilation (trop and pheny), topical anaesthesia, speculum, Retcam3 (ant seg + 5 retinal images), handheld slit lamp	Ophthalmologist	Comprehensive eye examination by the same ophthalmologist
**Descriptive studies – red reflex testing**				
Baldino 2019	Red reflex test	Dilated; dark environment; indirect ophthalmoscope lens set at 0D; both eyes; at 45 cm.	Resident physicians (paediatrics)	Ophthalmologist with indirect ophthalmoscope before discharge
Cagini 2017	Red reflex test	Direct ophthalmoscope using standard methods	Paediatrician, neonatalogists	In nearest ophthalmology department; onward to tertiary centre if needed
Eventov-Friedman 2010	Red reflex test	Indirect ophthalmoscope set at 0 degrees	Resident physicians (paediatrics) & neonatologists	Not stated, but probably eye department in same hospital
Yazgan 2012	Red reflex test	Indirect ophthalmoscope in dark room	No details	Referred to an ophthalmologist
**Descriptive studies – wide-field digital imaging**				
Goyal 2018	Retinal imaging	Dilated, 130 degrees; RetCam; 5 fields	Optometrists	Images read by ophthalmology resident
Li 2013	Comprehensive eye exam	Undilated: torch (pupils, external eye, ant seg); Dilated: ant seg (RetCam), retinoscopy, hand held slit lamp, RR for media opacity; then wide field imaging (RetCam) 5 images	Ophthalmologist with assistant (and/or nurse)	Referral to ophthalmology clinic
Li 2017	Comprehensive eye exam	Undilated: torch (pupils, external eye; ant seg); Dilated: anterior segment examination (RetCam), wide field imaging (RetCam) 5 of images	Ophthalmologist with assistant	Further exam by ophthalmologist or referral to ophthalmology clinic
Ozkurt 2018	Comprehensive eye exam	Torch for external eye; RR using standard method with direct oph (no dilation)	Ophthalmologist	Same ophthalmologists
Tang 2018	Red reflex & wide field imaging	RR (no details ? with RetCam); Dilated wide field retinal imaging; 5 fields	Ophthalmologist with nurse assistant	Same ophthalmologist, or referred
Vinekar 2015	Retinal imaging	RetCam dilated anterior images; posterior segment video and images captured	Trained technician and nurse	Not clear who read images; ped retina specialist examined screen positive babies within 7 days

**Reported diagnostic test accuracy as well as the clinical findings of screening; NA = not applicable

Commented [JE2]: Seem to be 16 studies here not 15 as stated in the abstract
As above

Table S3. Included studies in relation to the comparisons and outcomes of the review

	Primary outcomes			Secondary outcomes		
	**Proportion of newborns identified by 8 weeks of age with clinically significant eye conditions (12 studies)**	**Age in months at clinical management of the eye condition (1 studies)**	**Outcome of management on mortality (retino-blastoma) or visual function (eye conditions) (0 studies)**	**Diagnostic test accuracy of the tests / devices used (1 study)**	**Adverse effects of eye screening (1 study)**	**Uptake of referral by infants who screen positive (0 studies)**
**Studies with comparison groups**						
Universal screening versus no screening (3 studies)	2 studies of congenital cataract (Sweden)	1 study: congenital cataract surgery before 42 days (Sweden)	-	-	1 study before and after introducing red reflex testing (Israel)	
Comparison of two different tests (1 studies)				1 study: red reflex testing vs imaging (China**)		
**Descriptive studies**						
Red reflex testing (4 studies)	4 studies (in Brazil, Israel, Italy, Turkey)	-				-
Imaging (6 studies)	6 studies (4 in China**, 2 jn India)	-				-

**One study in China reported two outcomes

Table S4a. Clinically significant eye conditions detected using red reflex testing

Author	Country/income(15)	Sample size	No of cases	Prevalence/ 100,000
**All significant conditions***				
Yazgan(9)	Turkey, upper middle	2,715	3	111
Eventov-Friedman(8)	Israel, high	11,500	5	44
Baldino(6)	Brazil, upper middle	11,833	4	34
Cagini(7)	Italy, high	22,272	3	14
**Cataract**				
Yazgan	Turkey, upper middle	2,715	2	75
Eventov-Friedman	Israel, high	11,500	4	35
Cagini	Italy, high	22,272	2	9
Baldino	Brazil, upper middle	11,833	1	9
**Retinoblastoma**				
Yazgan	Turkey, upper middle	2,715	1	39
Cagini	Italy, high	22,272	1	5
**Glaucoma**				
Baldino	Brazil, upper middle	11,833	2	20
**Other significant conditions***				
Eventov-Friedman	Israel, high	11,500	1	9
Baldino	Brazil, upper middle	11,833	1	9

Table S4b. Clinically significant eye conditions detected using widefield digital imaging

Author	Country/income(15)	Sample size	No of cases	Prevalence / 100,000
**All significant conditions***				
Li (2013)(11)	China, upper middle	3,573	32	896
Vinekar (14)	India, lower middle	1,021	9	882
Sun (5)	China, upper middle	7,641	57	746
Li (2017)(12)	China, upper middle	14,786	80	541
Goyal(10)	India, lower middle	1,152	4	347
Tang (1)	China, upper middle	196,108	379	193
**Cataract**				
Vinekar	As above	1,021	1	98
Li (2017)	As above	14,786	11	74
Li (2013)	As above	3,573	2	56
Sun	As above	7,641	2	26
Tang	As above	196,108	39	20
**Retinoblastoma**				
Vinekar	As above	1,021	1	98
Li (2013)	As above	3,573	2	56
Li (2017)	As above	14,786	3	20
Sun	As above	7,641	1	13
Tang	As above	196,108	5	3
**Glaucoma**				
Goyal	As above	1,152	2	174
**Other significant***				
Li (2013)	As above	3,573	28	784
Tang	As above	196,108	335	171
Sun	As above	7,641	53	694
Vinekar	As above	1,021	7	686
Li (2017)	As above	14,786	66	446
Goyal	As above	1,152	2	174

Appendix S5. Risk of bias in included studies

We assessed the risk of bias for included studies which provided comparative or diagnostic test accuracy data.

Comparative studies (ROBINS-1 checklist)

Potential source of bias	Magnusson 2003	Magnusson 2013	Ulanovsky 2015
Due to confounding	Serious	Serious	Serious
In selection of study participants	Low	Low	Low
Bias in classification of interventions	Low	Low	Low
Due to deviations from intended interventions	NI	NI	NI
Due to missing data	NI	Ni	NI
In measurement of outcomes	Moderate	Moderate	Moderate
In selection of reported result	Low	Low	Low
Overall risk of bias	Serious	Serious	Serious

NI: No information

Analytical cross-sectional studies (Joanna Briggs Institute checklist)

Potential source of bias	Ozkurt 2018	Tang 2018
Were criteria for inclusion in the sample clearly defined?	Yes	Yes
Were study subjects and the setting described in detail?	No	Yes
Was the exposure measured in a valid and reliable way?	No	Unclear
Were objective, standard criteria used to measure the condition?	Yes	Yes
Were confounding factors identified?	No	No
Were strategies to deal with confounding factors stated?	No	No
Were the outcomes measured in a valid and reliable way?	Yes	Unclear
Was appropriate statistical analysis used?	No	No
**Overall risk of bias**	**High**	**High**

Diagnostic test accuracy study (Joanna Briggs Institute checklist)

**Potential source of bias**	**Sun (2106)**
Was a consecutive or random sample of patients enrolled?	Yes
Was a case control design avoided?	Yes
Did the study avoid inappropriate exclusions?	Yes
Were index test results interpreted without knowledge of reference standard results?	Unclear
If a threshold was used, was it pre-specified?	NA
Is the reference standard likely to correctly classify the target condition?	Yes
Were reference standard results interpreted without knowledge of index test results?	No
Was there an appropriate interval between index test and reference standard?	No
Did all patients receive the same reference standard?	Yes
Were all patients included in the analysis?	Yes
**Overall risk of bias**	**High**

NA: Not applicable

Appendix S6. Scoping Guideline review

Nine guidelines were included (Figure 1). Four were national screening programme specifications (UK, Canada, New Zealand and India), three were ophthalmology/paediatric society guidelines (USA), one paediatric society guideline (Canada) and one WHO recommendation from a training package was identified on the WHO Europe Office website. Data from the latter were extracted from the online training module as the corresponding policy documents could not be identified despite online searches and contacting WHO.

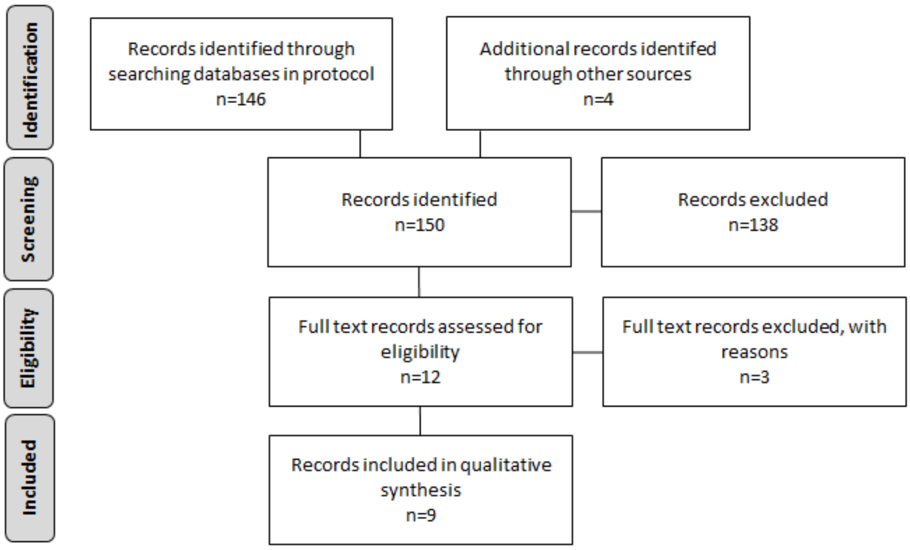

Figure: PRISMA chart for guideline review selection

Appendix S7. References of included studies

1. Tang H, Li N, Li Z, Zhang M, Wei M, Huang C, et al. Fundus examination of 199 851 newborns by digital imaging in China: a multicentre cross-sectional study. Br J Ophthalmol. 2018;102(12):1742-6.
2. Magnusson G, Jakobsson P, Kugelberg U, Lundvall A, Maly E, Tornqvist K, et al. Evaluation of screening procedures for congenital cataracts. Acta Paediatr. 2003;92(12):1468-73.
3. Magnusson G, Bizjajeva S, Haargaard B, Lundstrom M, Nystrom A, Tornqvist K. Congenital cataract screening in maternity wards is effective: evaluation of the Paediatric Cataract Register of Sweden. Acta Paediatr. 2013;102(3):263-7.
4. Ulanovsky I, Shnaider M, Geffen Y, Smolkin T, Mashiach T, Blazer S, et al. Performing red reflex eye examinations increases the rate of neonatal conjunctivitis. Acta Paediatr. 2015;104(12):e541-5.
5. Sun M, Ma A, Li F, Cheng K, Zhang M, Yang H, et al. Sensitivity and Specificity of Red Reflex Test in Newborn Eye Screening. J Pediatr. 2016;179:192-6 e4.
6. Baldino V, Eckert GU, Rossatto J, Wagner MB. Red reflex test at the maternity hospital: results from a tertiary hospital and variables associated with inconclusive test results. J Pediatr (Rio J). 2019.
7. Cagini C, Tosi G, Stracci F, Rinaldi VE, Verrotti A. Red reflex examination in neonates: evaluation of 3 years of screening. Int Ophthalmol. 2017;37(5):1199-204.
8. Eventov-Friedman S, Leiba H, Flidel-Rimon O, Juster-Reicher A, Shinwell ES. The red reflex examination in neonates: an efficient tool for early diagnosis of congenital ocular diseases. Isr Med Assoc J. 2010;12(5):259-61.
9. Hamza Yazgan AY, Esengül Keleş, Arzu Gebeşçi, Mehmet Demirdöven, Bülent Baştürk1, Selim Genç. Assessing the effectiveness of the red reflex test (Brückner) in early diagnosis of congenital eye disorders. Turk Arch Ped. 2012;47:163-4.
10. Goyal P, Padhi TR, Das T, Pradhan L, Sutar S, Butola S, et al. Outcome of universal newborn eye screening with wide-field digital retinal image acquisition system: a pilot study. Eye (Lond). 2018;32(1):67-73.
11. Li LH, Li N, Zhao JY, Fei P, Zhang GM, Mao JB, et al. Findings of perinatal ocular examination performed on 3573, healthy full-term newborns. Br J Ophthalmol. 2013;97(5):588-91.
12. Li LH, Wu WC, Li N, Lu J, Zhang GM, Zhao JY, et al. Full-Term Neonatal Ophthalmic Screening in China: A Review of 4-Year Outcomes. Ophthalmic Surg Lasers Imaging Retina. 2017;48(12):983-92.
13. Ozkurt Z, Balsak S, Yildirim Y, Yuksel H, Caca I. Associations between red reflex abnormality, consanguinity and intensive care hospitalization of newborns in Turkey. East Mediterr Health J. 2018;24(7):631-6.
14. Vinekar A, Govindaraj I, Jayadev C, Kumar AK, Sharma P, Mangalesh S, et al. Universal ocular screening of 1021 term infants using wide-field digital imaging in a single public hospital in India - a pilot study. Acta Ophthalmol. 2015;93(5):e372-6.
15. Bank W. World Bank Country and Lending Groups. [Available from: https://datahelpdesk.worldbank.org/knowledgebase/articles/906519-world-bank-country-and-lending-groups.
